# A systematic review of the psychological and social benefits of participation in sport for adults: informing development of a conceptual model of health through sport

**DOI:** 10.1186/1479-5868-10-135

**Published:** 2013-12-07

**Authors:** Rochelle M Eime, Janet A Young, Jack T Harvey, Melanie J Charity, Warren R Payne

**Affiliations:** 1 Institute of Sport, Exercise and Active Living, Victoria University, PO Box 14428, Melbourne, Victoria 8001, Australia; 2 School of Health Sciences, University of Ballarat, PO Box 663, Ballarat, Victoria 3353, Australia

**Keywords:** Sport, Health, Psychological, Psychosocial, Social

## Abstract

**Background:**

The definition of health incorporates the physical, social and mental domains, however the Physical Activity (PA) guidelines do not address social health. Furthermore, there is insufficient evidence about the levels or types of PA associated specifically with psychological health. This paper first presents the results of a systematic review of the psychological and social health benefits of participation in sport by adults. Secondly, the information arising from the systematic review has been used to develop a conceptual model of Health through Sport.

**Methods:**

A systematic review of 14 electronic databases was conducted in June 2012, and studies published since 1990 were considered for inclusion. Studies that addressed mental and/or social health benefits from participation in sport were included.

**Results:**

A total of 3668 publications were initially identified, of which 11 met the selection criteria. There were many different psychological and social health benefits reported, with the most commonly being wellbeing and reduced distress and stress. Sport may be associated with improved psychosocial health in addition to improvements attributable to participation in PA. Specifically, club-based or team-based sport seems to be associated with improved health outcomes compared to individual activities, due to the social nature of the participation. Notwithstanding this, individuals who prefer to participate in sport by themselves can still derive mental health benefits which can enhance the development of true-self-awareness and personal growth which is essential for social health. A conceptual model, Health through Sport, is proposed. The model depicts the relationship between psychological, psychosocial and social health domains, and their positive associations with sport participation, as reported in the literature. However, it is acknowledged that the capacity to determine the existence and direction of causal links between participation and health is limited by the cross-sectional nature of studies to date.

**Conclusion:**

It is recommended that participation in sport is advocated as a form of leisure-time PA for adults which can produce a range of health benefits. It is also recommended that the causal link between participation in sport and psycho-social health be further investigated and the conceptual model of Health through Sport tested.

## Introduction

It is important to participate regularly in physical activity (PA) to improve the likelihood of living a healthy life. To assist people living a healthy life, there are specific PA guidelines [1]. These guidelines include recommendations to avoid inactivity given any activity is better than being sedentary and even low levels of participation are associated with some health gains. However for a substantial health gain to be realised it is recommended that adults participate in PA for at least 150 minutes per week of moderate-intensity, or 75 minutes a week of vigorous-intensity aerobic PA or an equivalent combination of both moderate and vigorous PA [1].

Recommended minimum levels of PA were historically based on identified quantitative relationships between PA and physical health benefits [2]. Although mental health benefits have been referenced in more recent guidelines, to date ‘insufficient evidence precludes conclusions about the minimal or optimal types or amounts of physical activity for mental health’ [2] (Part G Section 8 p39). Instead of specifying a recommended level of PA for mental health benefits, mental health is often assessed in relation to the existing PA recommendations based on physical health benefits [3-7].

The World Health Organisation’s (2006) definition of health incorporates three domains, physical, mental, and social [8]. However, social health is not incorporated into the PA guidelines. Notwithstanding this, the literature informing the PA guidelines does suggest that social support through participation in PA can contribute to positive mental health aspects [2].

There are many different ways that people can be physically active. During peoples leisure-time is one way. Within the context of leisure-time PA there are different participation modes, settings and types of PA [9]. Eime et al., (2013) have distinguished four modes of leisure-time PA: team sport, individual sport, organised but non-competitive PA; and non-organised PA [9]. Sport is a popular form of leisure-time PA. Participation in sport is often in a social context. Because of this social nature, it is conjectured that sport participation may be associated with greater psychosocial health benefits than other forms of PA [10].

In a previous study, a Health through Sport model was developed for children and adolescents [11]. This study concluded that there were many different psychological and social health benefits reported, with the most common being improved self-esteem and improved social interaction/integration, followed by fewer depressive symptoms [11]. The authors concluded that for children and adolescents, sport may be associated with improved psychosocial health benefits above and beyond improvements attributed to participation in general PA [11]. The aim of this systematic review was to investigate the psychological and social benefits of participation in sport for adults, and to investigate the applicability of the Health through Sport model to adults.

## Methods

The criteria for considering studies for this review were as follows, and as reported in [11].

Inclusion criteria were

1. Studies published in English between Jan 1990 and May 2012 inclusive.

2. Original research or reports published in peer review journals or government or other organisational publications which reported primary data.

3. Studies which presented data that addressed mental and/or social health benefits from participation in sport. In this context, the following definitions were adopted: ‘sport’ - “a human activity of achieving a result requiring physical exertion and/or physical skill which, by its nature and organisation, is competitive and is generally accepted as being a sport” [12]. ‘health’ – “a state of complete physical, mental and social well-being and not merely the absence of disease and infirmity” [8]; ‘mental’ - “of or referring to the mind or to the processes of the mind, such as thinking, feeling, sensing, and the like” [13] (p475) ‘mental health’ – “Mental Health refers to a broad array of activities directly or indirectly related to the mental well-being component included in the WHO’s definition of health…It is related to the promotion of well-being, the prevention of mental disorders, and the treatment and rehabilitation of people affected by mental disorders” [14,15] ‘social’: “Relating to the interactions of individuals, particularly as members of a group or a community ” [13] (p475); ‘social health’: “That dimension of an individual’s well-being that concerns how he gets along with other people, how other people react to him, and how he interacts with social institutions and societal mores.” [16] (p 152). In this study, we also used the following terms: ‘psychological’ – synonymous with those aspects of ‘mental’ that do not include the treatment and rehabilitation of people affected by mental disorders’; and ‘psychosocial’ - “…any situation in which both psychological and social factors are assumed to play a role” [17] (p638).

4. Studies where the data pertained to the individual level (i.e. for persons versus communal or national level).

Exclusion criteria were:

1. Studies or reports that addressed ‘exercise’, ‘physical activity’, ‘physical education’, or ‘recreation’, and not sport. Definitions of these terms are: ‘Exercise’ –“physical activity that is planned, structured, repetitive, and purposive in the sense that improvement or maintenance of one or more components of physical fitness is an objective” [15] (p128); ‘Physical activity’ - “bodily movement produced by skeletal muscles that results in energy expenditure” [15] (p126); ‘Physical education’ - “a sequential, developmentally appropriate educational experience that engages students in learning and understanding movement activities that are personally and socially meaningful, with the goal of promoting healthy living” [18] (p8); ‘Recreation’ – “pleasurable activity” [19] (p. 915).

2. Research/reports that addressed participation in ‘adapted’ sports (i.e. sport participation for persons with a physical and/or intellectual disability, such as wheelchair tennis).

3. Research/reports that addressed sub-populations subject to specific risks (i.e. studies with heroin users, ‘at risk’ individuals etc.).

4. Research/reports that addressed rehabilitation from, or management of, injury or illness.

5. Research/reports that addressed spectators, coaches or sports administrators.

6. Research/reports that addressed elite sports participants.

7. Research/reports that addressed ‘sport development’ programs that have an educational objective.

8. Book chapters, abstracts, dissertations and conference proceedings.

### Search methods for identification of studies, reports and publications

A systematic search of 14 electronic databases (AUSPORT, AusportMed, CINAHL, Cochrane Library, EBSCHOHost Research Databases, Health Collection, Informit, Medline Fulltext, PsycARTICLES, Psychology and Behavioral Sciences Collection, PsycINFO, PubMed, Scopus, SPORTDiscus Fulltext) was conducted in June 2012. We also consulted with the Australian Sports Commission to search the National Sports Information Centre records in order to identify relevant reports, publications and research not located through the search of the electronic databases cited above. Further, we conducted an internet search using the Google Scholar search engine (http://www.googlescholar.com) to locate additional studies in the Medicine, Social Sciences, Arts and Humanities subject areas. The Google Scholar search engine was also used to search for recognised International, National and State reports and publications that directly addressed the topic under consideration.

To search the electronic databases a combination of keywords and search terms was adopted. These key words and search terms were formulated by the authors of this systematic review as those they considered directly addressed the topic under consideration. These keywords and search terms constituted four groups, namely:

Group 1: sport

Group 2: health

Group 3: value, benefit, effect, outcome

Group 4: psychology, depression, stress, anxiety, happiness, mood, quality of life, social health, social relations, well, social connect, social functioning, life satisfaction, mental health, sociology, social.

Accordingly where possible, the database searches consisted of key words from Group 1 AND Group 2 AND Group 3 AND Group 4. The truncation symbol was added to the most basic word stem for each keyword to ensure all associated terms were included in the search.

### Study selection

**Figure 1:**
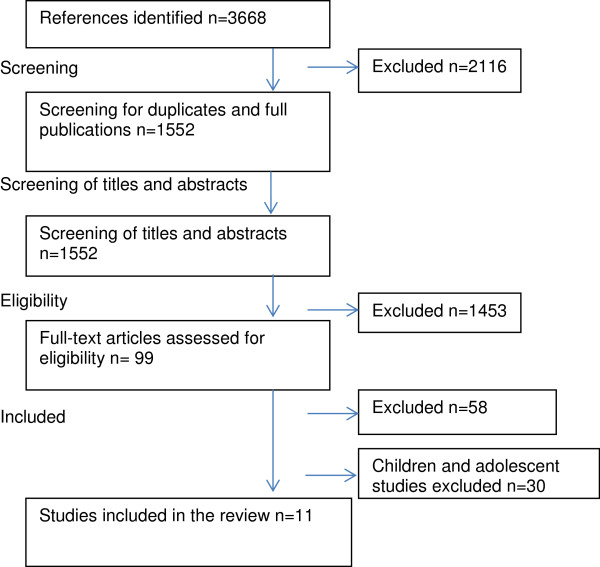
Stages of study selection.

Figure 1 provides a summary of the stages of study selection. Titles and abstracts of potentially relevant articles were screened by JY. Authors, JY and RE examined all full-text articles, and assessed the studies to ensure that they met the inclusion criteria. Any discrepancies were resolved through discussion between the two reviewers. Consensus was obtained for all included articles. After reviewing the selected studies it was decided that, given the breadth and complexity of the research domain, that studies focusing on children and adolescent would be further reviewed separately from studies focusing on adults. This review focuses on adults only. Studies that stated that they investigated adults’ sport participation were included. A separate systematic review focusing on children and adolescents has been published [11].

### Data collection and analysis

Data extracted from each of the studies included: study design and methodology; sample size; country of origin; age of participants; cohort of participants; gender of participants; study aim; sport variable; other PA variables; theoretical construct; key findings in relation to psychological and social health outcomes.

### Assessment of study quality

Study quality was objectively appraised using the Downs and Black checklist [20] This checklist has been used in other systematic reviews within the physical activity and health field [21,22]. This checklist includes 27 items grouped into categories: reporting (10), external validity (3), internal validity - bias (7), internal validity – confounding (6), and power (1). Twenty five items are scored as 1 (compliance) or 0 (non-compliance or inability to determine compliance); one item about confounding is scored as 2 (full compliance), 1 (partial compliance) or 0 (non-compliance or inability to determine compliance); and the item concerning power is scored (via a more complex algorithm) on a scale of 0–5.

Because most of the studies we reviewed did not involve interventions, a number of the items on the Downs and Black checklist were not generally applicable. We substituted a simpler power item (presence or absence of reference to a power analysis), and scored all items as 0, 1 or NA (not applicable). We calculated a summary quality score for each paper (except the two qualitative papers for which only five items were applicable) by expressing the number of compliant items as a percentage of the number of applicable items. We included these scores (ranging from 44% to 93%) in Table 1, and used the insights we gained through the scoring process in our discussion of study quality.

**Table 1:** Studies investigating the psychological and social health benefits of participation in sport for adults

**Reference**	Design*	Method**	**Sample (n)**	**Country**	**Age (yrs)**	Cohort***	Sex****	**Aim**	**Sport**	**Other PA**	**Theory**	**Key Finding(s)**	**Psychosocial Outcomes**	**Score (%)**
[23]	Quant.	Cross.	1427	Belgium	20-65	Adult	B	Analysis of the relationship between sports participation and stress	Favourite sports	no sport, other sports/PA	Mindful Movement theory	Little difference in perceived stress and emotional distress existed in men and women across different sports. Significant associations were found between participation in walking and meditation sports with stress appraisal and emotional distress among women.	Less stress and distress, less emotional distress (males) and increased social support	69
[24]	Quant.	Pros. Cross.	6751	Germany	18-45	Adult	B	Analysis of the effects of sport participation in long-term labour market variables, health and subjective well-being	Sport at least monthly	Less than monthly participation in sport	Nil	Positive mental health effects of sports participation included vitality, social functioning and role emotion. Sport has positive effects on health and subjective well-being.	Vitality, social functioning, role emotion, subjective well-being	87
[25]	Qual.	Cross.	14	Australia	16-25	Adol. and adult	M	Explore connections between sport and civic engagement	Sport	Nil	Phenomenological framework Grounded theory	Sport participants reported mental and health benefits including feeling good, confident, ability to cope with hard times and a sense of belonging.	Feeling good, confidence, coping with hard times, sense of belonging, resilience	67
[26]	Quant.	Cross.	16,627	England	16 yrs and older	Adol. and adult	B	Investigate impact of sports participation on subjective well-being	Sport	No sport and non-social interaction sports	Subjective wellbeing	Sports participation positively associated with subjective well-being.	Well-being/happiness	56
[27]	Qual.	Cross.	20	USA	17-23	Adol. and adult	B	Investigate mechanisms for creating sense of community within a sport setting	College athletes	Nil	Sense of community. Grounded theory, Phenomenoligcal approach	Five key factors (leadership opportunities, social spaces, competition, equity in administrative decisions, administrative consideration) were identified that fostered a sense of community within a collegiate sport context. Sense of community went beyond their sporting experience.	Sense of community	71
[10]	Quant.	Cross.	818	Australia	M 34-47	Adult	F	Examine health-related quality of life and life satisfaction in different forms of PA	Club sport	Walking and gymnasium	Nil	Women involved in club sport reported higher levels of mental well-being and life satisfaction compared with women engaged in the individual-based activities of walking and going to a gymnasium (ie participation in sport is associated with better mental well-being than other forms of PA). Club sport participants had better physical role functioning, vitality, social functioning and mental health.	Mental health, life satisfaction, vitality, social functioning	81
[28]	Quant.	Long.	30	Australia	M 24	Adult	M	Monitor changes in stress and recovery for Rugby League players	Rugby League	nil	Nil	Significant decreases in social stress were reported between weeks 1 and 4.	Less social stress	56
[29]	Quant.	Cross.	19,842	UK	M 45	Adult	B	Examine association between mental health and PA behaviours	Leisure Time Sport	Walking, Domestic PA	Nil	All types of PA associated with lower risk of psychological distress, with strongest effect observed for sport.	Less psychological distress	93
[30]	Quant.	Cross.	791	USA	M 20	Adult	B	Examined relationships among dimensions of athletic involvement (team sport, individual sport, athlete identity, jock identity)	Team sport	Individual sport and no sport	Nil	Participation in a team sport was associated with lower depression scores. Athlete identify mediated the relationship between team sport participation and depression.	Lower depression score	73
[31]	Quant.	Cross.	1919	Belgium	20-65	Adult	B	Examine associations between 5 types of PA with different contents: housework, leisure active transport, biking to/from work, walking to/from work and sports participation and mental health	Sport	Housework, leisure transport, walking and bike to work	Nil	Sports participation was the only type of PA inversely associcated with both stress and distress.	Less stress and distress	75
[32]	Quant.	Pros.	118	UK	M 21	Adult	B	Test hypotheses that importance of ratings of life aspirations would mediate the effects of participation in sport on psychological well-being	Competitive sport participation	Recreation sport participation	Self-Determination Theory	Recreational athletes reported higher psychological well-being than competitive athletes. The moral worth of sport does not reside so much in the frequency with which individuals engage in sport but in the goals and values people express through their participation.	Hedonic enjoyment and eudemonia wellbeing	44

*Quant. (Quantitative): Qual (Qualitative).

**Cross. (Cross-sectional): Pros. (Prospective): Long: (Longitudinal).

***Adol (Adolescent).

****M (Male): F (Female): B (Both Male and Female).

### Conceptual model development

**Figure 2:**
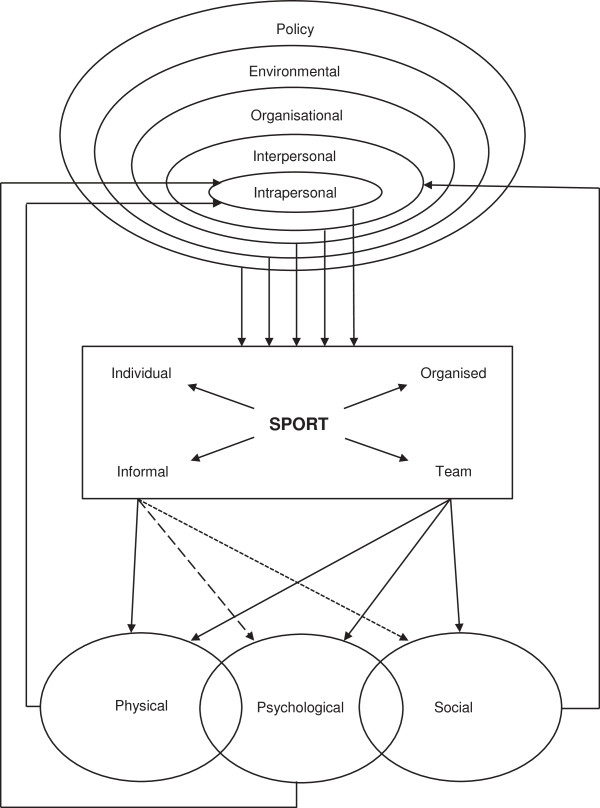
Health through sport conceptual model.

Based upon the literature presented in this review, and an accompanying review of literature regarding children and adolescents [11], a conceptual model of Health through Sport has been developed (Figure 2). The model depicts the relationship between determinants driving sport participation and the reported psychological and social health benefits of participation. The terminology used in this conceptual model is as defined in the inclusion criterion 3 above. The determinants are represented as per the Socio-Ecological Model [33,34]. Upon reviewing the studies, two dimensions of sport participation were identified, and it became evident that some reported health benefits were more likely to be associated with some contexts of sport participation than others. Therefore, a model was developed to represent the two contextual dimensions of sport participation and the different strengths of association between different contexts of sport participation and the three health aspects (physical, psychological and social).

With regard to causality, we note that most studies have been cross-sectional and observational in nature, and hence do not provide strong evidence of causality. The literature suggests that sport can have positive health benefits; however it is also the case that better health may predispose people to initiate and maintain participation in sport. A few longitudinal studies provide stronger evidence of causality. However, in the absence of randomised and controlled experimental studies, which are challenging to implement in this domain, it will remain difficult to unequivocally determine the nature and direction of causality. Notwithstanding this, terms like ‘outcome’ and ‘benefit’ of sport participation have been used to describe the results of many of the studies reviewed, and we have used the same terminology in reviewing these studies.

## Results

A total of 3668 publications were initially identified. Table 1 provides a summary of the 11 studies that met the inclusion criteria. The majority of studies were quantitative (n = 8) rather than qualitative (n = 3). There were no randomised-controlled trials in the identified studies, with the majority being cross-sectional (n = 8). Two were classified as prospective. One of these was a study using cross-sectional sample data over a 22 year period [24] and the other states it is prospective without providing details of waves of measurement time [32]. Whilst one study is classified as longitudinal, it only covers a single Rugby League season with multiple measurement periods within the season [28].

The sample sizes in the studies that met the inclusion criteria ranged from 14 to 19,842 participants. The majority of studies (n = 8) had over 100 participants. The majority of studies were conducted in Australia (n = 3) and United Kingdom (n = 3), followed by United States of America (n = 2), Belgium (n = 2), and Germany (n = 1). The age of study participants was generally adult exclusively (n = 7) with four studies including older adolescents too. Nearly all (n = 8) included both males and females. The three Australian studies focused exclusively on males [25,28] or females [10].

Most studies scored highly on the modified Downs and Black scale of study quality (median 71 percent; range 44–93 percent). Those studies scored above the median value (higher quality) were all cross-sectional quantitative studies [10,24,29-31]. None of the studies of higher quality incorporated a theoretical approach. Within the studies of higher quality, there was no consistency regarding the components of sport and other PA investigated. Instead, these included: sport at least monthly [24], club sport [10], leisure time sport [29], team sport [3], and sport [31]. As a consequence of the varied study designs, low number of studies, and differing aspects of sport investigated, there was no clear distinction between the key findings of the higher and lower ranked studies in terms of the psychological and social health benefits of participation in sport.

Thirteen different psychosocial health aspects were identified in the ten studies (Table 2). The most common positive health benefit was improved well-being [24,25,28,32], followed by reduced stress [23,28,31], reduced distress [23,29,32] and increased social functioning [10,24] and vitality [10,24].

**Table 2:** Summary of the psychosocial health aspects associated with sport participation for adults

**Category**	**Specific health aspect**	**Study**
Psychological	Well-being	[24,25,28,32]
Psychological	Reduced stress	[23,28,31]
Psychological	Reduced distress	[23,29,31]
Social	Social functioning	[10,24]
Psychological	Vitality	[10,24]
Psychological	Hedonic enjoyment	[32]
Psychological	Subjective wellbeing	[26]
Psychosocial	Belonging	[25]
Psychological	Life satisfaction	[10]
Psychological	Lower depression	[30]
Psychological	Mental health	[10]
Psychosocial	Role emotion	[24]
Psychosocial	Sense of community	[27]

The definitions and measurement of sport participation varied considerably. Several studies classified sport participation by frequency only [24,26,29,31], whilst others classified measurement by favourite sport [23], club sport [10], team sport [30] or context of participation such as competitive versus recreational [32]. The types of sport activities were generally not defined, however Eime et al. [10] defined club participants as participating in netball and/or tennis, and King et al. [28] measured stress and recovery in Rugby League. The measurement was often in binary terms where those participating in sport were compared to those participating in other activities or to those not participating in any activity. Sport was not always the focus of each paper and in some instances sport was measured as one context of PA participation [31].

Half of the studies incorporated a theoretical perspective. Two incorporated a Grounded theory theoretical perspective to their enquiry [25,27], whilst others utilised specific theories such as the Mindful Movement theory and Self-Determination theory to explain behaviours.

Few differences were evident between the conclusions of studies of higher and lower quality or of different study design. Although the total number of studies reviewed was relatively small, there were some differences in the reported health outcomes associated with different contexts of participation. Therefore the following presents a summary of the psychological and social health benefits of participation in sport according to the different contexts of sport participation/definition.

### Sport participation in general

A study by Asztalos et al. [31] compared sport to other forms of PA with regard to mental health. As these authors explain, the relationship between physical activity and mental health may change across different domains of activity. In their study Asztalos et al. examined the associations between participation in five domains of PA: housework leisure active transportation, biking to/from work, walking to/from work, and sports participation [31]. The aspects of mental health measured were perceived stress and psychological distress. The mean frequency of sports participation in the sample of 1919 adults was once per week, averaging 2.75 hours/week. Sport participation was based upon this mean, and specifically the adults had to participate weekly for at least 2.75 hours. Whilst the data was controlled for gender, age and occupation, they did not do so for level of PA. Nonetheless, sports participation was the only type of PA inversely associated with both stress and distress. On the other hand, housework was associated with more stress and distress for women with blue-collar jobs. Biking to work was associated with more stress for men with blue-collar jobs. The authors provided some reasons why they believe that sport participation, and not other types of activities, had better mental health outcomes. They explained that sport represents a chosen leisure-time activity and aims for recreation, enjoyment and social interaction which promotes well-being. Furthermore, these improved levels of well-being are not associated with PA that implies compulsion which to an extent, housework and active transport do. Similarly, Harmer et al. [29] investigated PA in different domains of sport, walking and domestic PA. They found significant benefits of participation on reduced psychological distress for all types of activity with participants only needing a minimum of 20 per week to have significant differences in mental health measures [29]. The strongest effects were observed for sport participation. A dose–response pattern was reported, which demonstrated greater risk reduction with higher volume and/or intensity of participation.

### Club sport participation

A recent study investigated sport participation more specifically. Eime et al. [10] hypothesised that sports club participants would have improved health related quality of life (HRQoL) and life satisfaction given the social nature of their participation, compared to more individual PA activities such as walking and going to the gymnasium [10]. These authors also compared these contexts of participation with a normative reference group of participants, which measured the same health aspects. Even after adjusting for differences in levels of PA, club participants had better physical role functioning, vitality, social functioning, mental health and life satisfaction than gymnasium and walking participants. These results support the notion that participation in a socially engaged manner can contribute to mental health and life-satisfaction. The authors concluded that the improved health benefits in the sport club group compared to individual based PA may result from enhanced social connectedness, social support, peer bonding and self-esteem which may be provided by club support [10].

Similarly, Miller et al. investigated group (defined as team in the literature) compared to individual sport participation in terms of depression and suicidal behaviour [30]. Team sport was defined as requiring two or more people on the same side to coordinate their movements. Miller et al. found that both participation in team sport and athlete identify were associated with lower depression scores. Specifically, athletic identify mediated the relationship between sport participation and depression.

### Recreational and competitive sport

Within club and/or team sport participation there can be different contexts of play, including competitive and recreation participation. One study investigated the contributions of recreational sport and competitive sport to life aspirations and psychological well-being [32]. The prospective design meant that demographic characteristics and participation in recreational versus competitive sport were collected at baseline, and after two weeks, and general psychological well-being was assessed. The life aspirations had a mediating effect on the relationship between participation in recreational and competitive sport and psychological well-being. Furthermore, recreational participants showed a preference for intrinsic life aspirations compared with competitive participants and reported higher psychological well-being [32]. The researchers concluded that the relative importance of intrinsic over extrinsic aspirations is a key dimension in predicting psychological well-being. They proposed that their findings are in line with the Self-Determination theory [35] given intrinsic motivation is related to personal growth, community contributions, health and meaningful relationships and is far more rewarding and enhances eudemonia and enjoyment compared with extrinsic life aspirations. Furthermore, Chatzisarantis and Hagger argued that, because the focus of recreational participation is not so much on winning compared to competitive sport structures, participants in recreational sport place greater emphasis on intrinsic versus extrinsic life aspirations.

Only one study investigated a single sport being Rugby League [28]. These researchers investigated Rugby League participation across a season and changes in social stress, and how this related to fatigue and injury. There were significant differences observed in social stress between weeks 1 and 4. On the recovery scales significant differences were observed for social recovery between weeks 1 and 5 and general well-being between weeks 2 and 3 [28].

### Other sport participation

Warner and Dixon (2011) interviewed former college athletes and asked them about mechanisms for creating a sense of community within a sport setting. The study found that sense of community was mainly fostered by administrative consideration, leadership opportunities, equity in administrative decisions, competition and social spaces [27].

Two studies had a less defined measure of sport, with favourite sport [23] and frequency of participation [24] being used. In the more recent study, sport participation was defined as how often individuals participated in their favourite sport per week [23]. The authors presented results of frequency of participation to demographics but not to the health outcomes of stress appraisal and emotional distress. However, they did provide detailed information on the types of sports and the associated health outcomes. Sport type-related differences relative to stress appraisal and emotional distress were scarce. However, sport versus no sport participation was associated with significantly less stress and distress. Therefore the differences in the PA to mental health relationship are insignificant as long as the individual’s participation is in the personally favoured types of sport. The authors concluded that, in trying to explain the PA - mental health relationship, previous research has narrowed the spectrum of PA domains down to sport participation. As a result Asztalsos et al. proposed that ‘no one activity fits all recommendations’ in relation to stress appraisal and emotional distress. However they did emphasise the fact that there is an association between specific types of sports and individual preferences, and that it is important for people to choose a sport that suits them best [23]. In particular, individuals who prefer to participate in sport in solitary modes can experience mental health benefits from this chosen form of participation. Specifically, they concluded that such modes of participation can enhance the development of true-self-awareness and personal growth, which is important to social health [23].

Lastly, Lechner conducted a micro econometric study analysing the effects of sport participation on long-term labour market variables, as well as health and subjective well-being indicators [24]. In addition to the sizeable positive long-term labour market effects in terms of earnings and wages, there were positive effects on health and subjective well-being. The researchers measured sport participation in terms of frequency ranging from at least every week to none. The health measures included the degree of disability, perceived health status and general satisfaction with their health status. Sport was positively associated with all health outcomes.

### Conceptual model

A conceptual model of Health through Sport is proposed (Figure 2) that is based on three primary categories of outcome as per [11]: physical, psychological and social; and two secondary categories: physical/psychological – aspects involving both the physical and psychological elements, and psychosocial – aspects involving both psychological and social elements.

While our model incorporates all five categories and thus depicts the full range of health aspects, the ‘physical’ aspects have been well reviewed elsewhere [2] and so this paper in focused on the psychological and social aspects, as defined above.

As previously described [11], the model includes three major elements: (a) determinants of sports participation, (b) sport itself, and (c) health outcomes of sport participation. The ‘determinants’ element is based on the well-established social ecological model [33,34] and is represented as concentric rings spreading out from the individual’s intrapersonal characteristics to widening spheres of influence. The sport element incorporates two dimensions of context: individual – team and informal – organised, each of which is almost dichotomous, but also has some intermediate variants (e.g. running alone, running in an informal group, running for a club team, running in a club relay team). The three types of health outcomes - physical, psychological and social - are shown as overlapping, representing the fact that there may be interactions and interrelationships between physical and psychological aspects and between psychological and social health aspects. For example, there are relationships between physical fitness and mental state; and interpersonal relationships may satisfy needs for belongingness and, as such, influence psychological health. Another example is resilience, whereby psychological health may influence an individual’s capacity to engage in interpersonal relationships.

The different strengths of the various linkages between the sport element and the health outcomes represent the notion that all forms of sport contribute strongly to physical health. However, while organised and/or team forms also contribute strongly to psychological and social outcomes, informal and/or individual forms contribute somewhat less to psychological outcomes and relatively little to social outcomes. Finally, we have noted the limited evidence of causality in the literature reviewed. This ambiguity or reciprocity could perhaps be represented by double-headed arrows linking the physical, psychological and social elements to the sport element, but we have represented it by ‘feedback loops’ from the three outputs to the intrapersonal and interpersonal determinants.

The structure of the model proposed is sufficiently general to be applicable both in the adult context and in the context of children and adolescents [11]. Each element and each link shown in Figure 2 is applicable in both contexts. However, the specific details of each element or link may differ between the adult and child/adolescent contexts. The relative importance of specific determinants of participation (within the five social-ecological domains) is not the same for children/adolescents and adults; for example, schools are important organisational settings for sport participation among children and adolescents, but not for adults. The mechanisms of the links between sport participation and psychological and social health also differ at different stages of life; for example, developmental issues are important aspects of the psychological and social health domains for children and adolescents, but not for adults. Also, the feedback loops whereby health status affects intrapersonal and interpersonal determinants of participation are different in detail at different life stages. The main differences between the health outcomes of the children and adolescent compared to adults were that the children and adolescent studies highlighted mainly social health aspects and the adults more psychological. Improved social interaction/integration and social skills and improved self-esteem were the most common health aspects reported in the systematic review for children and adolescents [11]. Whereas the most common health aspects reported in this study related to psychological health aspects of well-being, and reduced stress and distress.

### Limitations

This systematic review has some limitations. Whilst the search strategy, based on a-priori inclusion and exclusion criteria, was comprehensive and encompassed grey literature which reported primary data, conference proceedings were not included. Nor were non-English language articles included. The studies reviewed included a wide range of aims, focuses, measurement tools and indicators of both sport participation and health outcomes. This diversity of focus and methodology limited the extent of synthesis and precluded meta-analysis. Most studies were cross-sectional and used self-report measures. Therefore results should be interpreted with caution, and any conclusions regarding causation are conjectural.

## Conclusion

Whilst the number of studies investigating the psychological and social health benefits of sport participation for adults was not large, there was a general consensus that there are many psychological and social health benefits associated with participation in sport for adults. Furthermore, there is consistent evidence that club-based and team-based sport participation, when compared to other individual forms of PA, is associated with better psychological and social health outcomes. It is generally concluded that it is the social nature of this participation that is the factor mediating the relationship between participation and improved health. Furthermore, the concept of choice and fun seems to be a contributing factor to improved health. When people play a sport of their choice, it is fun and enjoyable in the social context and they are often intrinsically motivated to participate. In saying this, it is important for each individual to choose their sport so that it suits their preferences. Some individuals prefer to participate in sport in solitary modes and this participation can enhance their mental health. This in turn can contribute to the development of true-self-awareness and personal growth which is also significant for social health. In contrast to sport participation, other forms of PA, such as domestic chores and transport, are not necessarily enjoyable. The improved health aspects from sport participation were also often associated with recreational play rather than with competition.

In light of the research evidence, and acknowledging that research to date is predominantly based on cross-sectional studies, it is recommended that participation in sport is advocated as a form of leisure-time PA for adults which can produce a range of health benefits. It is also recommended that the causal link between participation in sport and psycho-social health be further investigated and the conceptual model of Health through Sport tested.

## Abbreviations

PA: Physical activity.

## Competing interests

The authors declare that they have no competing interests.

## Authors’ contributions

RME contributed to the study design, the review of literature, analysis of literature, model conceptualisation, manuscript conceptualisation and preparation. JAY contributed to the study design, the review of literature, analysis of literature, model conceptualisation, manuscript conceptualisation and preparation. JTH contributed to analysis of literature, model conceptualisation and representation, and manuscript preparation. MJC contributed to analysis of study quality and critical review of the manuscript. WRP contributed to the study design and critical review of the manuscript. All authors read and approved the final manuscript.
